# Supplementary material for: Two-colour single-molecule photoinduced electron transfer fluorescence imaging microscopy of chaperone dynamics
Source: Nat Commun. 2021 Nov 29;12:6964. doi: 10.1038/s41467-021-27286-5 (PMC8630005; doi:10.1038/s41467-021-27286-5)
Supplement: Supplementary file 1 — Supplementary Information [file 41467_2021_27286_MOESM1_ESM.pdf]

Supplementary Information

Two-colour single-molecule photoinduced electron transfer fluorescence imaging microscopy of chaperone dynamics

Jonathan Schubert[1], Andrea Schulze[1,2], Chrisostomos Prodromou[3] & Hannes Neuweiler[1,*]

[1]Department of Biotechnology and Biophysics, Julius-Maximilians-University Würzburg, Am Hubland, 97074 Würzburg, Germany

[2]Current address: Proteros Biostructures, Bunsenstr. 7a, 82152 Martinsried, Germany

[3]Biochemistry and Biomedicine, School of Life Sciences, University of Sussex, Falmer, Brighton BN1 9QG, United Kingdom

*Corresponding author: hannes.neuweiler@uni-wuerzburg.de

Supplementary Tables

Supplementary Table 1: Kinetics of closure determined using two-colour smPET fluorescence.

Reporter combination	Reporter[a]	a_1	τ_1 (s)	a_2	τ_2 (s)	$\tau_{mean\#}$ (s)	τ_{mean} (s)	k_c (min^{-1})
Lid^{Atto542}-NM^{JF646}	Lid^{Atto542}-1	0.17	29	0.83	287	244	195±41	0.31±0.07
	Lid^{Atto542}-2	1	143	-	-	143		
	Lid^{Atto542}-3	0.27	19	0.73	264	198		
	NM^{JF646}-1	1	86	-	-	86	100±27	0.60±0.16
	NM^{JF646}-2	1	137	-	-	137		
	NM^{JF646}-3	0.35	16	0.65	109	77		
DS^{Atto542}-NM^{JF646}	DS^{Atto542}-1	0.51	10	0.49	81	45	42±6	1.43±0.19
	DS^{Atto542}-2	0.24	4	0.76	44	34		
	DS^{Atto542}-3	1	46	-	-	46		
	NM^{JF646}-1	0.40	8	0.60	62	41	44±3	1.37±0.11
	NM^{JF646}-2	0.42	12	0.58	64	42		
	NM^{JF646}-3	1	48	-	-	48		
DS^{Atto542}-Lid^{JF646}	DS^{Atto542}-1	0.44	12	0.56	70	45	41±12	1.47±0.44
	DS^{Atto542}-2	0.48	4	0.52	100	54		
	DS^{Atto542}-3	0.46	5	0.54	41	24		
	Lid^{JF646}-1	0.65	6	0.35	91	35	137±150[b]	0.44±0.48[b]
	Lid^{JF646}-2[b]	0.28	17	0.72	478	349		
	Lid^{JF646}-3	0.44	6	0.56	42	26		
Lid^{Atto542}-A2W-NM^{JF646}	Lid^{Atto542}-1	1	98	-	-	98	85±12	0.71±0.10
	Lid^{Atto542}-2	1	87	-	-	87		
	Lid^{Atto542}-3	0.54	12	0.46	137	69		
	A2W-NM^{JF646}-1	1	101	-	-	101	67±26	0.90±0.34
	A2W-NM^{JF646}-2	1	59	-	-	59		
	A2W-NM^{JF646}-3	0.51	7	0.49	76	40		

[a]: the number (-#) denotes the measurement number.
a_n and τ_n denotes the relative amplitude and time constant of the n-th exponential decay.
(-): not determined.
$\tau_{mean\#}$: mean time constant of the decay calculated as the mean of the time constants τ_1 and τ_2 weighted by their relative amplitudes.
τ_{mean}: mean time constant calculated from $\tau_{mean\#}$ of measurements 1-3.
[b]: the large time constant and correspondingly large error of this measurement arose from an unusually large time constant τ_2 determined from the bi-exponential fit to the data of measurement #2.
k_c: mean rate constant of closure calculated from τ_{mean} ($k = 1/\tau_{mean}$).
Errors are ±s.d. of three measurements.

Supplementary Table 2: Kinetics of opening determined using two-colour smPET fluorescence.

Reporter combination	Reporter[a]	a_1	τ_1 (s)	a_2	τ_2 (s)	$\tau_{mean\#}$ (s)	τ_{mean} (s)	k_o (min^{-1})
$Lid^{Atto542}$-NM^{JF646}	$Lid^{Atto542}$-1	1	13	-	-	13	12±6	5±3
	$Lid^{Atto542}$-2	0.87	1	0.13	22	4		
	$Lid^{Atto542}$-3	1	19	-	-	19		
	NM^{JF646}-1	1	13	-	-	13	11±5	6±3
	NM^{JF646}-2	0.87	1	0.13	21	4		
	NM^{JF646}-3	1	16	-	-	16		
$DS^{Atto542}$-NM^{JF646}	$DS^{Atto542}$-1	1	13	-	-	13	13±2	4.6±0.5
	$DS^{Atto542}$-2	1	11	-	-	11		
	$DS^{Atto542}$-3	1	15	-	-	15		
	NM^{JF646}-1	1	13	-	-	13	12±1	4.8±0.5
	NM^{JF646}-2	1	11	-	-	11		
	NM^{JF646}-3	1	14	-	-	14		
$DS^{Atto542}$-Lid^{JF646}	$DS^{Atto542}$-1	1	13	-	-	13	15±4	4±1
	$DS^{Atto542}$-2	1	12	-	-	12		
	$DS^{Atto542}$-3	0.60	7	0.40	43	21		
	Lid^{JF646}-1	1	17	-	-	17	15±2	4.1±0.6
	Lid^{JF646}-2	1	12	-	-	12		
	Lid^{JF646}-3	0.47	4	0.53	24	15		
$Lid^{Atto542}$-A2W-NM^{JF646}	$Lid^{Atto542}$-1	1	14	-	-	14	15±2	4.1±0.6
	$Lid^{Atto542}$-2	0.52	4	0.48	22	13		
	$Lid^{Atto542}$-3	1	18	-	-	18		
	A2W-NM^{JF646}-1	1	16	-	-	16	15±1	3.9±0.2
	A2W-NM^{JF646}-2	0.56	8	0.44	22	14		
	A2W-NM^{JF646}-3	1	16	-	-	16		

[a]: the number (-#) denotes the measurement number.
a_n and τ_n denotes the relative amplitude and time constant of the n-th exponential decay.
(-): not determined.
$\tau_{mean\#}$: mean time constant of the decay calculated as the mean of the time constants τ_1 and τ_2 weighted by their relative amplitudes.
τ_{mean}: mean time constant calculated from $\tau_{mean\#}$ of measurements 1-3.
k_o: mean rate constant of opening calculated from τ_{mean} ($k = 1/\tau_{mean}$).
Errors are ±s.d. of three measurements.

Supplementary Figures

Supplementary Fig. 1

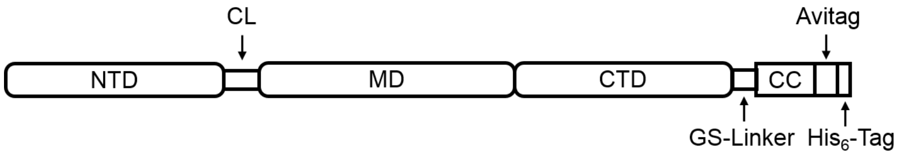

Supplementary Fig. 1: **Scheme of the yeast Hsp90 sequence modified at the C-terminus for sm immobilization**. The sequence area is shown from the N-terminus to the C-terminus from left to right. The N-terminal domain (NTD), charged linker (CL), middle domain (MD), and C-terminal domain (CTD) are indicated. The C-terminus was extended by a coiled-coil sequence (CC) separated by a GS-rich linker. C-terminal Avitag and His_6-tag are indicated.

Supplementary Fig. 2

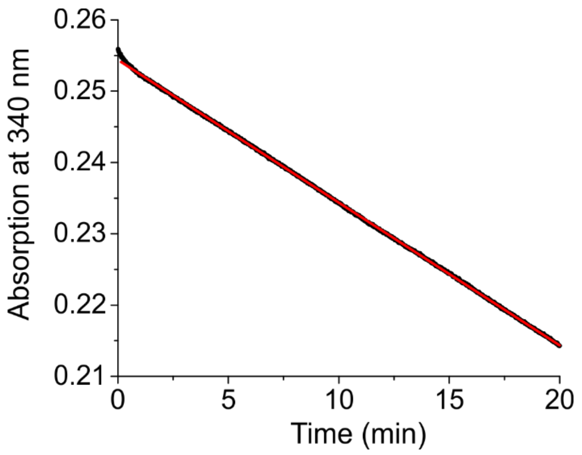

Supplementary Fig. 2: **Enzyme-coupled ATPase assay of the WinZipA2/WinZipB1 hetero-dimer of Hsp90**. NADH absorbance signal measured at 340 nm as a function of time during ATPase activity of hetero-dimeric yeast Hsp90 modified with WinZipA2 and WinZipB1 coiled-coil sequences at the C-terminus, at 25 °C. The red line is a linear fit to the data. Source data are provided as a Source Data file.

Supplementary Fig. 3

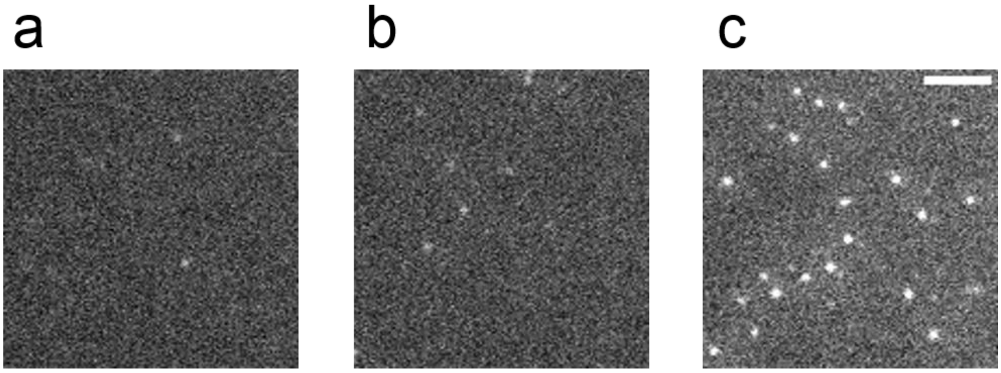

Supplementary Fig. 3: **Assessment of specificity of Hsp90 protein immobilization**. TIRF images (laser excitation at 640 nm) of (**a**) a DDS-modified quartz glass coverslip; (**b**) a DDS-modified coverslip after treatment with Tween 20 and biotinylated BSA, followed by incubation with 1 nM AttoOxa11-modified Hsp90 (no Neutravidin treatment); and (**c**) a DDS-modified coverslip, after treatment with Tween 20 and biotinylated BSA, followed by treatment with Neutravidin, and subsequent incubation with 1 nM AttoOxa11-modified Hsp90 (scale bar: 2 µm). Experiments were repeated three times with similar results.

Supplementary Fig. 4

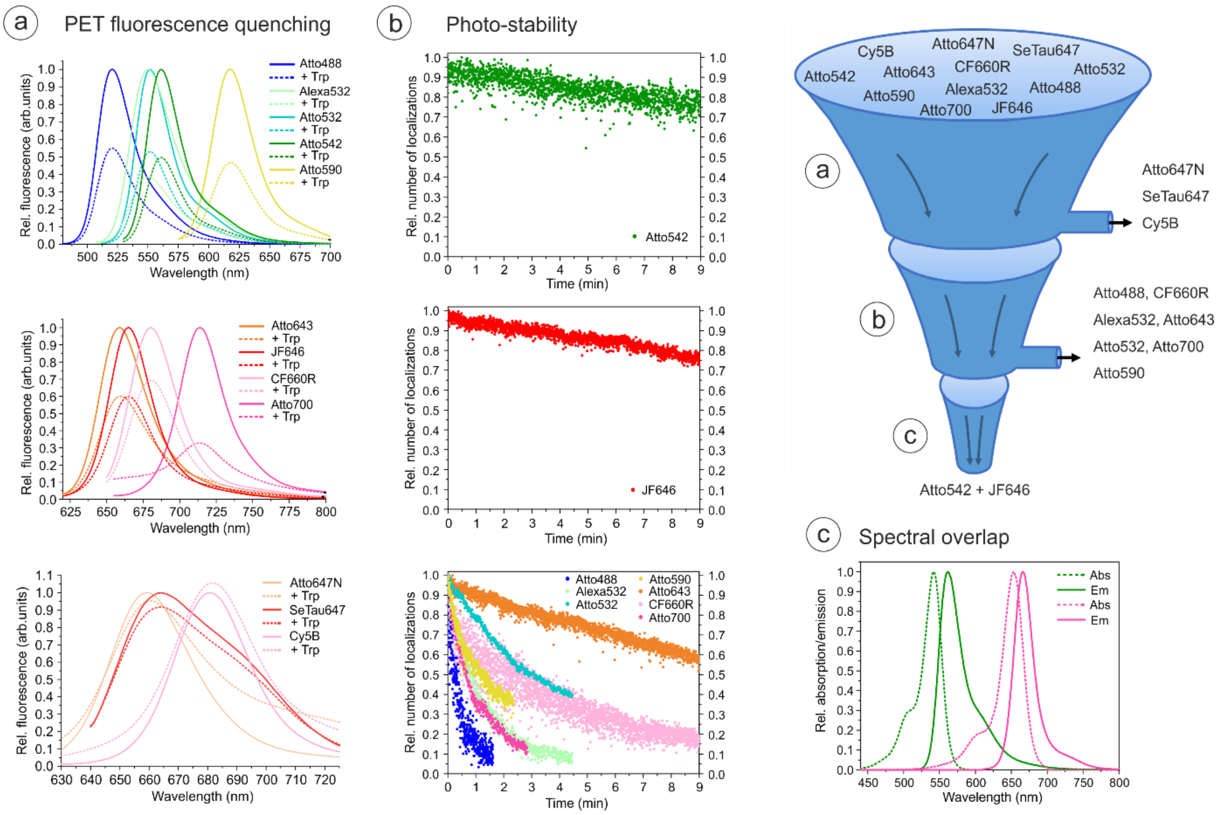

Supplementary Fig. 4: **Screening and selection of fluorophores for two-colour smPET fluorescence microscopy.** Twelve commercial fluorophores were tested. (**a**) First, quenching of fluorescence by Trp through PET was tested. To this end, steady-state fluorescence emission spectra of 150 nM fluorophore in the absence (solid lines) and in the presence (dashed lines) of 25 mM Trp were recorded. The discarded fluorophores that exhibited weak or no quenching are shown in the lowest panel. (**b**) Second, photo-stability during single-molecule detection was tested. To this end, individual fluorophore-Hsp90 conjugates immobilized on glass surface supports (as described in Methods) were measured under oxygen-depleted solution using TIRF microscopy. The decrease of the number of fluorescent spots over time, recorded in series of TIRF images, was a measure of photo-stability. (**c**) Atto542 and JF646 were most photo-stable and further exhibited little spectral overlap. The procedure of fluorophore selection is illustrated by the funnel on the right hand side. Importantly, both Atto542 and JF646 showed little fluorescence fluctuations in sm time traces, contrasting AttoOxa11 (**Fig. 3a-d** in the paper), and were thus considered most suitable for two-colour smPET fluorescence experiments. Source data are provided as a Source Data file.

Supplementary Fig. 5

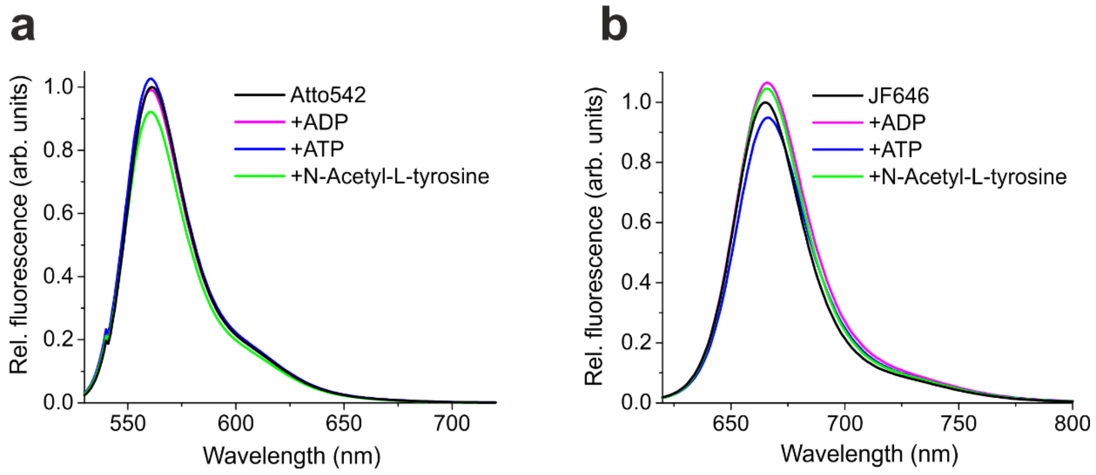

Supplementary Fig. 5: **Influence of ATP, ADP and tyrosine on fluorescence of Atto542 and JF646**. (**a**), (**b**) Steady-state fluorescence emission spectra of 150 nM Atto542 (**a**) and JF646 (**b**), both shown in black, and measured in presence of 25 mM ADP (magenta), 25 mM ATP (blue) and 25 mM N-acetyl-L-tyrosine (green). Spectra are normalized to the maximal value of fluorescence emission intensity of the labels. The N-acetyl derivative of L-tyrosine was applied because of the low water-solubility of L-tyrosine. Source data are provided as a Source Data file.

Supplementary Fig. 6

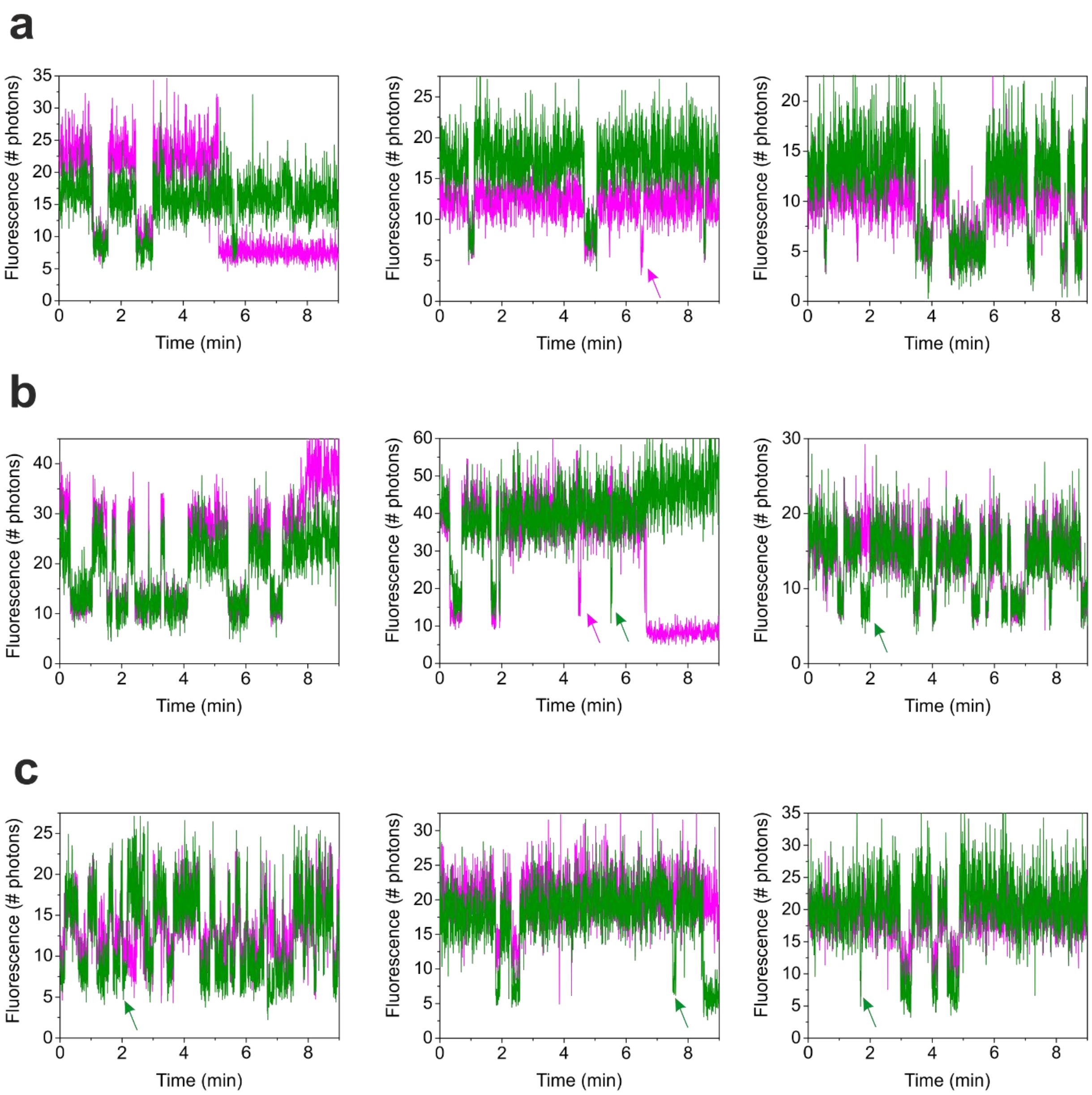

Supplementary Fig. 6: **Two-colour smPET fluorescence microscopy of Hsp90 dynamics in the presence of ATP**. Fluorescence intensity time traces of Hsp90 constructs Lid^{Atto542}-NM^{JF646} (**a**), DS^{Atto542}-NM^{JF646} (**b**), and DS^{Atto542}-lid^{JF646} (**c**), recorded in the presence of 4 mM ATP. Fluorescence emission intensities of Atto542 and JF646 are shown in green and magenta, respectively. Non-synchronous fluctuations are indicated by arrows. Off-states that persisted until the end of time traces indicate photo-bleaching. Source data are provided as a Source Data file.

Supplementary Fig. 7

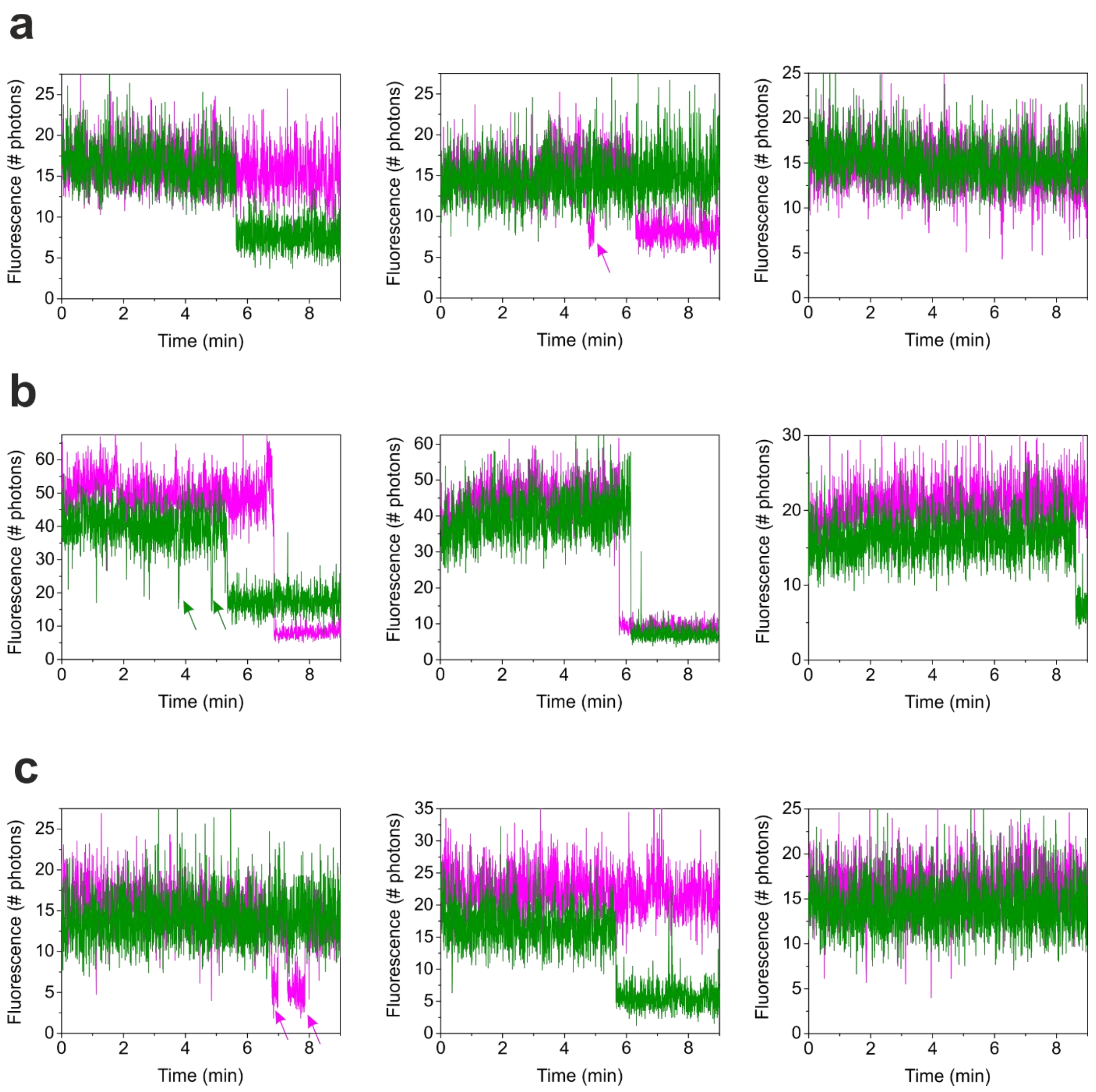

Supplementary Fig. 7: **Two-colour smPET fluorescence microscopy of Hsp90 dynamics in the absence of ATP**. Fluorescence intensity time traces recorded from the Hsp90 constructs Lid^{Atto542}-NM^{JF646} (**a**), DS^{Atto542}-NM^{JF646} (**b**), and DS^{Atto542}-lid^{JF646} (**c**). Fluorescence emission intensities of Atto542 and JF646 are shown in green and magenta, respectively. Non-synchronous fluctuations are indicated by arrows. Off-states that persisted until the end of time traces indicate photo-bleaching. Source data are provided as a Source Data file.

Supplementary Fig. 8

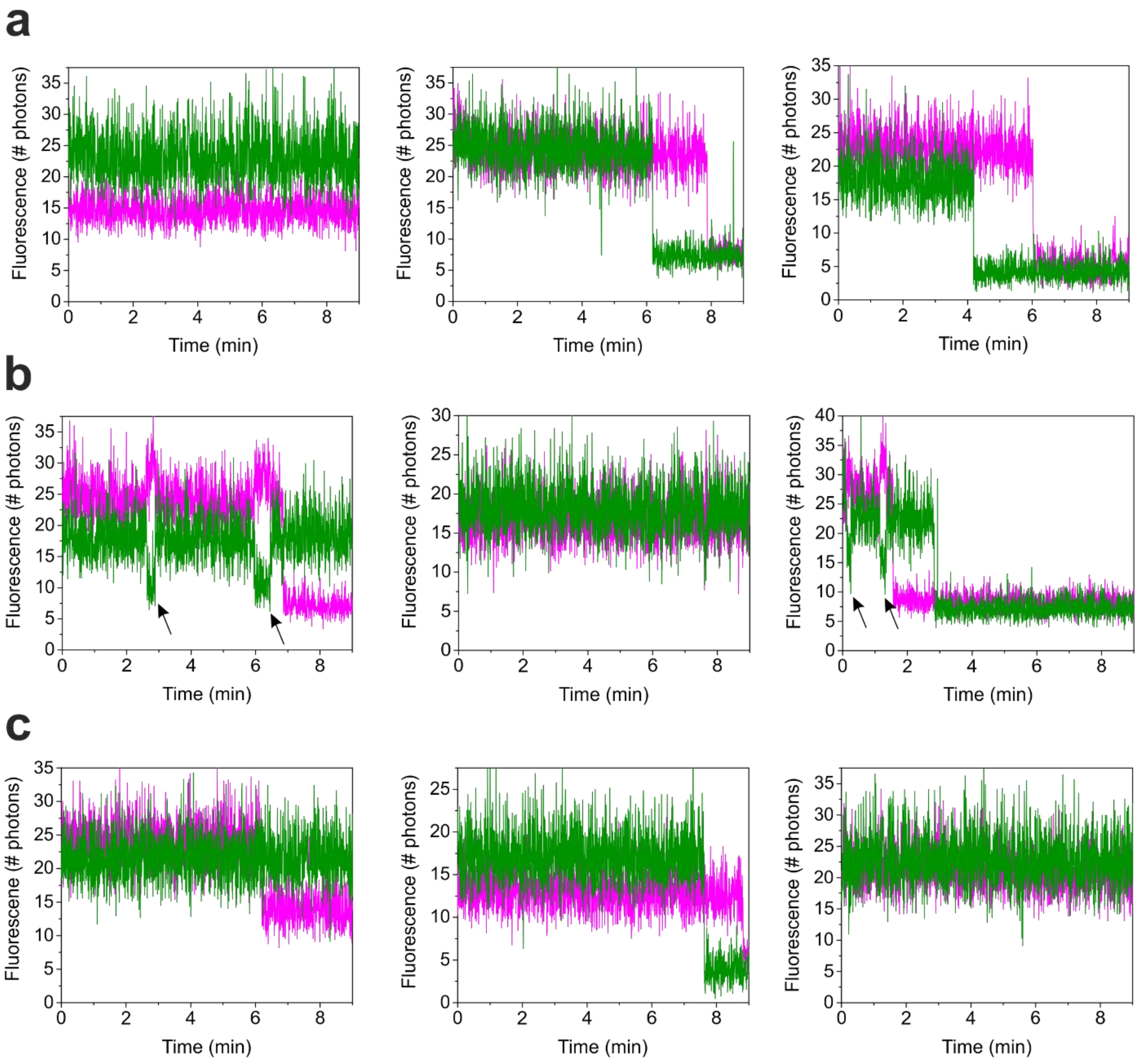

Supplementary Fig. 8: **Two-colour smPET fluorescence microscopy of Hsp90 construct** $\mathbf{DS^{Atto542\text{-}noTrp}\text{-}NM^{JF646\text{-}noTrp}}$. Fluorescence intensity time traces recorded from construct $DS^{Atto542\text{-}noTrp}\text{-}NM^{JF646\text{-}noTrp}$ that lacks mutations E162W and N298W are shown. (**a, b**) Representative time traces measured in the presence of 4 mM ATP. (**c**) Representative time traces measured in the absence of ATP. Fluorescence emission intensities of Atto542 and JF646 are shown in green and magenta, respectively. FRET events, i.e., anti-correlated changes of fluorescence emission intensities of Atto542 and JF646, are indicated by arrows. Off-states that persisted until the end of time traces indicate photo-bleaching. Source data are provided as a Source Data file.

Supplementary Fig. 9

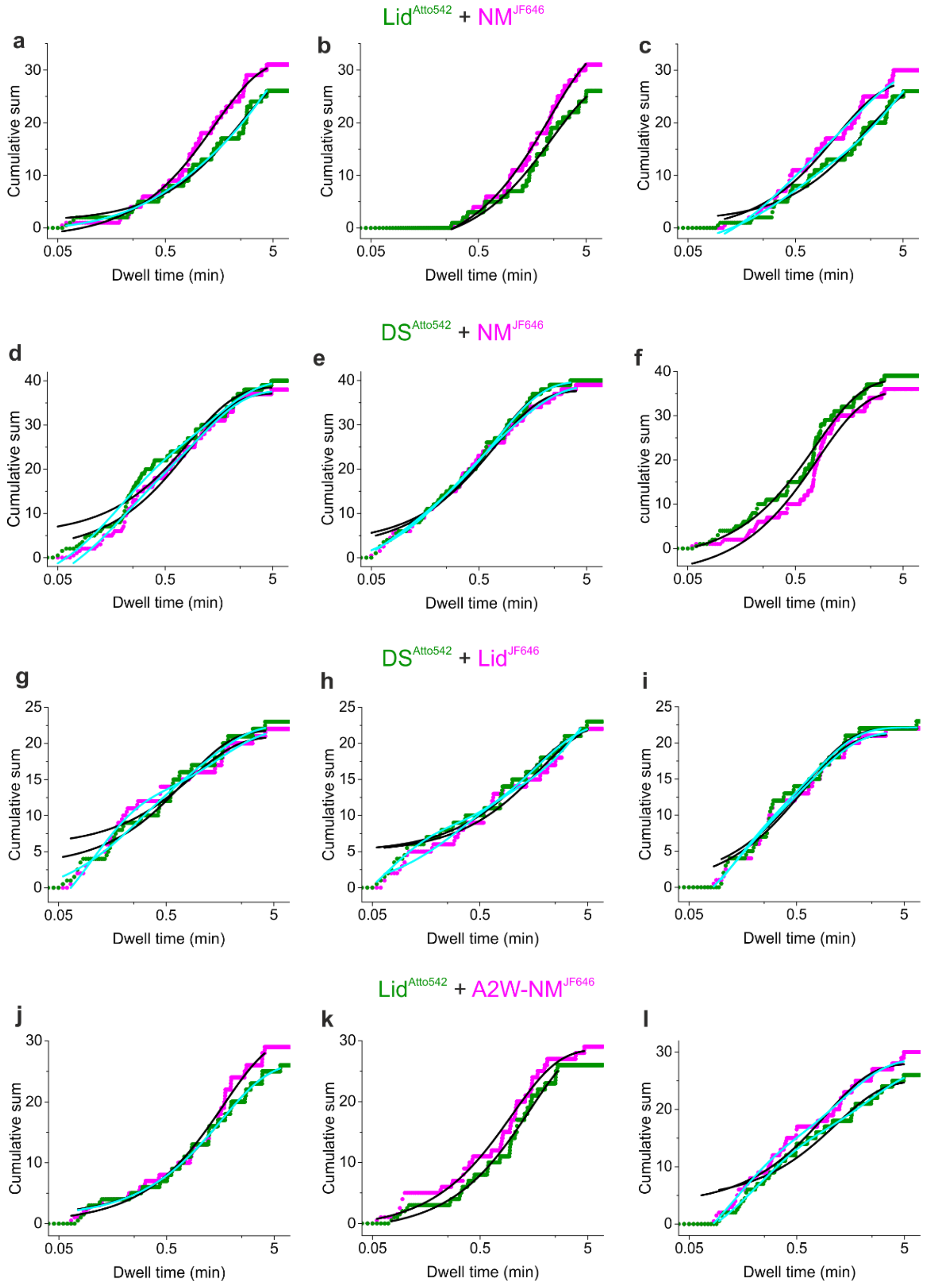

Supplementary Fig. 9: Kinetics of closure of local structural elements of Hsp90 detected using two-colour smPET fluorescence. Cumulative sum plots of dwell times in fluorescent states determined from three independent measurements of the reporter pairs $Lid^{Atto542}$-NM^{JF646} (**a-c**), $DS^{Atto542}$-NM^{JF646} (**d-f**), $DS^{Atto542}$-Lid^{JF646} (**g-i**) and $Lid^{Atto542}$-A2W-NM^{JF646} (**j-l**), in the presence of ATP. Black lines are mono-exponential fits to the data. Cyan lines are bi-exponential fits to the data. Source data are provided as a Source Data file.

Supplementary Fig. 10

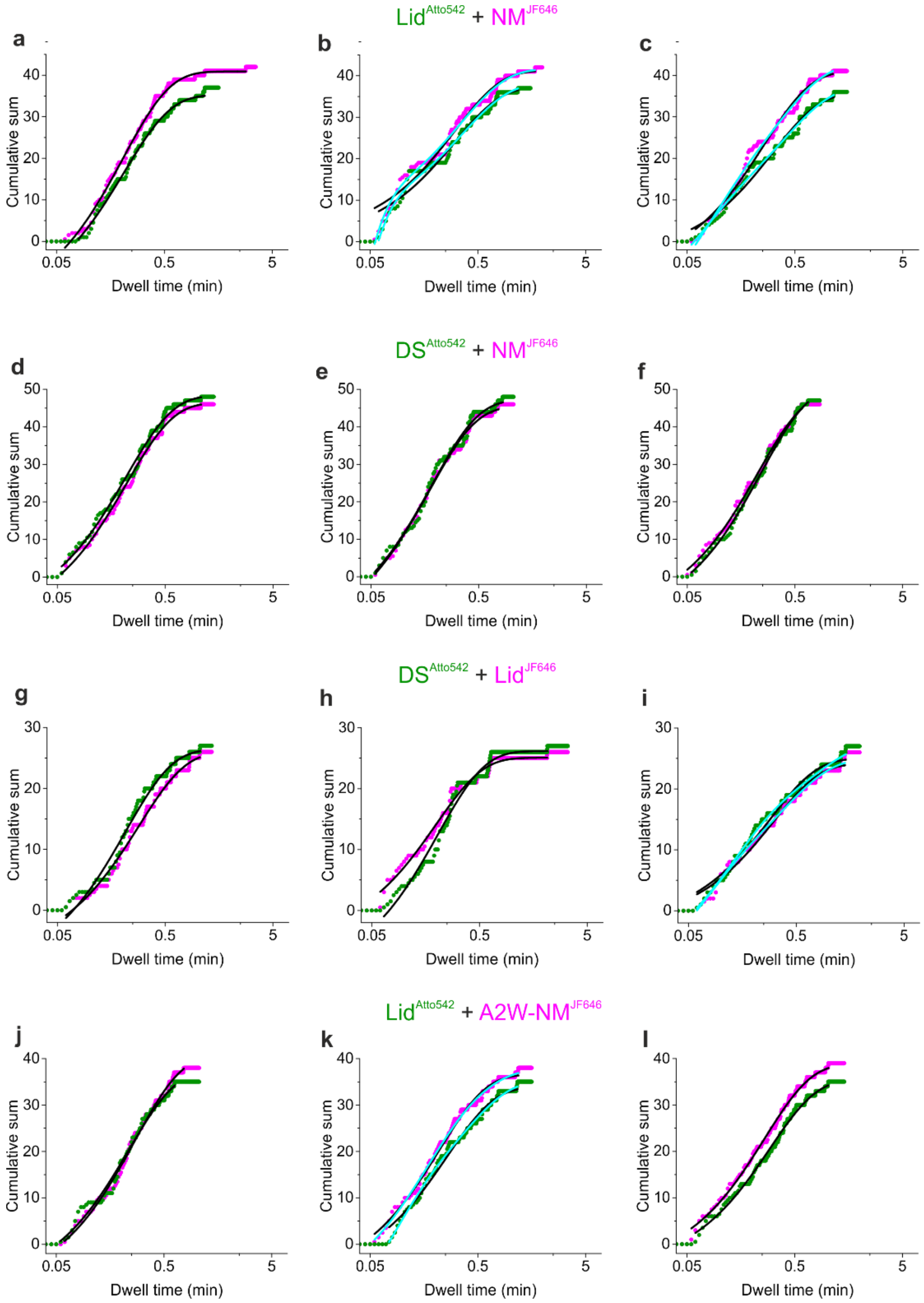

Supplementary Fig. 10: **Kinetics of opening of local structural elements of Hsp90 detected using two-colour smPET fluorescence**. Cumulative sum plots of dwell times in fluorescence-quenched states determined from three independent measurements of the reporter pairs Lid^{Atto542}-NM^{JF646} (**a-c**), DS^{Atto542}-NM^{JF646} (**d-f**), DS^{Atto542}-Lid^{JF646} (**g-i**) and Lid^{Atto542}-A2W-NM^{JF646} (**j-l**), in the presence of ATP. Black lines are mono-exponential fits to the data. Cyan lines are bi-exponential fits to the data. Source data are provided as a Source Data file.

Supplementary Fig. 11

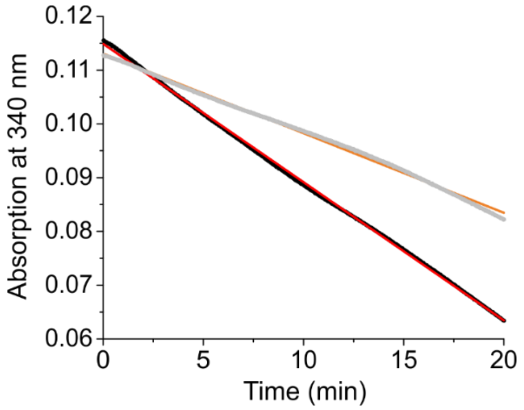

Supplementary Fig. 11: **Enzyme-coupled ATPase assay of wild-type Hsp90 and mutant A2W**. NADH absorbance signal measured at 340 nm as a function of time during ATPase activity of wild-type Hsp90 (grey) and mutant A2W (black) at 25 °C. The red and orange lines are linear fits to the data. Source data are provided as a Source Data file.

Supplementary Fig. 12

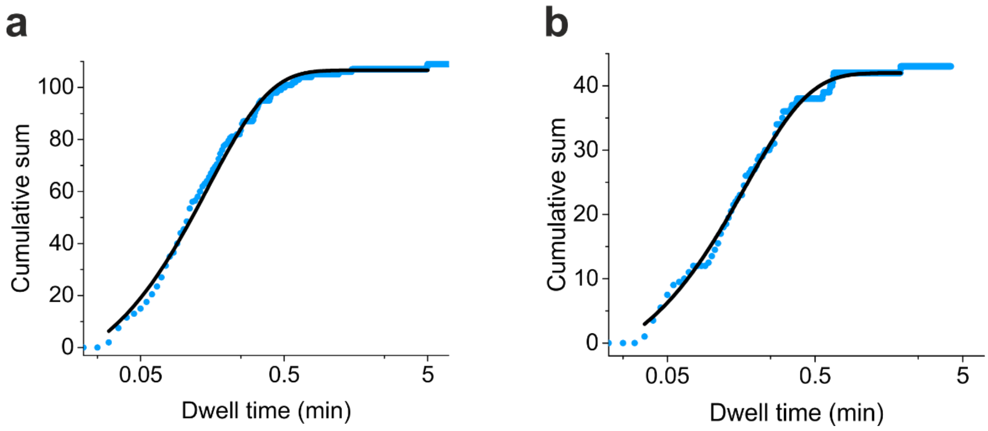

Supplementary Fig. 12: **Cumulative sum plots of dwell times in fluorescence-quenched states originating from non-synchronous transitions**. (**a**) Cumulative sum plot of dwell times in off-states caused by non-synchronous transitions measured in the presence of 4 mM ATP. (**b**) Cumulative sum plot of dwell times in off-states caused by non-synchronous transitions measured in the absence of ATP. Data were pooled from two-colour smPET fluorescence traces of the reporters $Lid^{Atto542}$-NM^{JF646}, $DS^{Atto542}$-NM^{JF646} and $DS^{Atto542}$-Lid^{JF646}. Black lines are mono-exponential fits to the data. Source data are provided as a Source Data file.
