# Supplementary material for: Two-colour single-molecule photoinduced electron transfer fluorescence imaging microscopy of chaperone dynamics
Source: Nat Commun. 2021 Nov 29;12:6964. doi: 10.1038/s41467-021-27286-5 (PMC8630005; doi:10.1038/s41467-021-27286-5)
Supplement: Supplementary file 2 — Reporting Summary [file 41467_2021_27286_MOESM2_ESM.pdf]

nature research

Corresponding author(s): Hannes Neuweiler

Last updated by author(s): Nov 11, 2021

# Reporting Summary

Nature Research wishes to improve the reproducibility of the work that we publish. This form provides structure for consistency and transparency in reporting. For further information on Nature Research policies, see our Editorial Policies and the Editorial Policy Checklist.

## Statistics

For all statistical analyses, confirm that the following items are present in the figure legend, table legend, main text, or Methods section.

| n/a | Confirmed | |
|---|---|---|
| ☐ | ☒ | The exact sample size ($n$) for each experimental group/condition, given as a discrete number and unit of measurement |
| ☐ | ☒ | A statement on whether measurements were taken from distinct samples or whether the same sample was measured repeatedly |
| ☒ | ☐ | The statistical test(s) used AND whether they are one- or two-sided<br>*Only common tests should be described solely by name; describe more complex techniques in the Methods section.* |
| ☒ | ☐ | A description of all covariates tested |
| ☒ | ☐ | A description of any assumptions or corrections, such as tests of normality and adjustment for multiple comparisons |
| ☐ | ☒ | A full description of the statistical parameters including central tendency (e.g. means) or other basic estimates (e.g. regression coefficient) AND variation (e.g. standard deviation) or associated estimates of uncertainty (e.g. confidence intervals) |
| ☒ | ☐ | For null hypothesis testing, the test statistic (e.g. $F$, $t$, $r$) with confidence intervals, effect sizes, degrees of freedom and $P$ value noted<br>*Give P values as exact values whenever suitable.* |
| ☒ | ☐ | For Bayesian analysis, information on the choice of priors and Markov chain Monte Carlo settings |
| ☒ | ☐ | For hierarchical and complex designs, identification of the appropriate level for tests and full reporting of outcomes |
| ☒ | ☐ | Estimates of effect sizes (e.g. Cohen's $d$, Pearson's $r$), indicating how they were calculated |

*Our web collection on statistics for biologists contains articles on many of the points above.*

## Software and code

Policy information about availability of computer code

| | |
|---|---|
| Data collection | Spectra Manager (Jasco, version 2); Solis (Version 4.28.30014, Andor, Oxford Instruments). |
| Data analysis | Fiji (version 1.53c), plugin BUnwarpJ version 2.6.13; rapidSTORM version 3.3; Origin2016G; Python 3.8, script 2CsmPET (available at: https://github.com/jsch111/2CsmPET). |

For manuscripts utilizing custom algorithms or software that are central to the research but not yet described in published literature, software must be made available to editors and reviewers. We strongly encourage code deposition in a community repository (e.g. GitHub). See the Nature Research guidelines for submitting code & software for further information.

## Data

Policy information about availability of data

All manuscripts must include a data availability statement. This statement should provide the following information, where applicable:

- Accession codes, unique identifiers, or web links for publicly available datasets
- A list of figures that have associated raw data
- A description of any restrictions on data availability

The raw data underlying Figures and Supplementary Information are provided as Source Data file. The raw smTIRF images underlying Figures 2 and 4 are provided at https://doi.org/10.5281/zenodo.5674994. Source data are provided with this paper.

# Field-specific reporting

Please select the one below that is the best fit for your research. If you are not sure, read the appropriate sections before making your selection.

- [x] Life sciences
- [ ] Behavioural & social sciences
- [ ] Ecological, evolutionary & environmental sciences

For a reference copy of the document with all sections, see nature.com/documents/nr-reporting-summary-flat.pdf

# Life sciences study design

All studies must disclose on these points even when the disclosure is negative.

| | |
|---|---|
| Sample size | Sample sizes were chosen such that mean kinetic quantities obtained from single-molecule data were larger than standard deviations of three independent measurements. The criterion was fullfilled in the analysis of fluorescence transitions from about 150 individual molecules per Hsp90 construct investigated. |
| Data exclusions | In two-colour experiments, fluorescence data of single Hsp90 molecules that contained only one fluorescence label (identified by a lack of co-localization of the two labels Atto542 and JF646) were discarded (no pre-established exclusion criteria were applied). |
| Replication | Reproducibilty was confirmed by three replicate measurements. |
| Randomization | Randomization was not relevant to this study because data recorded from single molecules are inherently stochastic in nature. |
| Blinding | Blinding was not relevant to this study for the reason given above. |

# Reporting for specific materials, systems and methods

We require information from authors about some types of materials, experimental systems and methods used in many studies. Here, indicate whether each material, system or method listed is relevant to your study. If you are not sure if a list item applies to your research, read the appropriate section before selecting a response.

## Materials & experimental systems

| n/a | Involved in the study | |
|---|---|---|
| x | | Antibodies |
| x | | Eukaryotic cell lines |
| x | | Palaeontology and archaeology |
| x | | Animals and other organisms |
| x | | Human research participants |
| x | | Clinical data |
| x | | Dual use research of concern |

## Methods

| n/a | Involved in the study | |
|---|---|---|
| x | | ChIP-seq |
| x | | Flow cytometry |
| x | | MRI-based neuroimaging |
