# Supplementary material for: Two-colour single-molecule photoinduced electron transfer fluorescence imaging microscopy of chaperone dynamics
Source: Nat Commun. 2021 Nov 29;12:6964. doi: 10.1038/s41467-021-27286-5 (PMC8630005; doi:10.1038/s41467-021-27286-5)
Supplement: Supplementary file 4 — Description of Additional Supplementary Files [file 41467_2021_27286_MOESM4_ESM.pdf]

Title: Supplementary Movie 1:
Description: Synchronicity of conformational changes within Hsp90 during closure of the molecular clamp. The position of the N-terminal domains in the open state is arbitrarily selected. Sub-millisecond dynamics of Lid and N-terminal β-strand detected using fluorescence correlation spectroscopy (ref. 29) are not represented in this movie.

Title: Supplementary Movie 2:
Description: Movie showing details of the Lid and DS conformational changes during dimerization of the N-terminal domains of Hsp90. The position of the N-terminal domains in the open state is arbitrarily selected. Atoms shown as spheres represent the position of the fluorophore (green, magenta) and the Trp (blue). The green/blue coloured pair monitors lid closure, while the magenta/blue coloured pair monitors DS. Fluorescence is quenched when Trp and fluorophore come into close contact. Sub-millisecond dynamics of Lid and N-terminal β-strand detected using fluorescence correlation spectroscopy (ref. 29) are not represented in this movie.

Title: Supplementary Movie 3:
Description: Movie showing details of the NM association during N-terminal dimerization of Hsp90. The position of the N-terminal domains in the open state is arbitrarily selected. Atoms shown as spheres represent the position of the fluorophore (orange) and Trp (blue) pair that monitors NM association. Fluorescence is quenched when Trp and fluorophore come into close contact. Sub-millisecond dynamics of Lid and N-terminal β-strand detected using fluorescence correlation spectroscopy (ref. 29) are not represented in this movie
